# What is known about Indigenous women’s dissatisfaction of Birthing experiences in mainstream maternity hospitals in Australia, Aotearoa, Canada, US, Kalaallit Nunaat and Sápmi? A systematic scoping review

**DOI:** 10.3389/fpubh.2025.1495197

**Published:** 2025-03-21

**Authors:** Nina Sivertsen, Tahlia Johnson, Grete Mehus, Tove Synnøve Mentsen Ness, Susan Smith, Josephine McGill

**Affiliations:** ^1^College of Nursing and Health Sciences, Caring Futures Institute, Flinders University, Adelaide, SA, Australia; ^2^Faculty of Health Sciences, UiT Arctic University of Norway, Tromsø, Norway; ^3^Centre for Sámi and Indigenous Studies, Faculty of Education and Art, Nord University, Levanger, Norway; ^4^Library Services, Flinders University, Adelaide, SA, Australia

**Keywords:** indigenous, birthing, dissatisfaction, hospital, mainstream, healthcare

## Abstract

**Introduction:**

Understanding Indigenous women’s dissatisfaction with birthing experiences is vital for improving maternal healthcare. It highlights the need for compassionate, respectful care that meets women’s physical and emotional needs. Addressing these concerns can enhance patient satisfaction, reduce postpartum mental health issues and wellness, and ensure safer, more positive outcomes for mothers and babies.

**Objectives:**

This scoping review aimed to identify what is known about Indigenous women’s dissatisfaction of birthing experiences in mainstream maternity hospitals.

**Inclusion criteria:**

This review considered primary research studies that reported on reasons for dissatisfaction of birthing experiences, and strategies implemented to improve quality of clinical practice around women’s dissatisfaction of birthing experiences in mainstream maternity hospitals in Australia, Aotearoa, Canada, US, Kalaallit Nunaat and Sápmi.

**Findings:**

A total of 22 manuscripts reporting 22 studies met the inclusion criteria and were included in the synthesis.

**Discussion:**

There is a need for culturally safe trauma informed care, inclusive communication, active decision-making involvement and greater inclusion of Indigenous perspectives in maternity care, including the involvement of Indigenous birth support workers where appropriate and inclusion of Birthing on Country models of care.

**Conclusion:**

This review reveals that the medicalisation and evacuation of Indigenous women for childbirth cause cultural, geographic, and social disconnection, despite infant safety benefits. It underscores the need for better cultural safety education, communication, and the inclusion of cultural practices in care, with support from Indigenous birth support workers being essential.

## Introduction

Maternal and perinatal outcomes of Indigenous women and infants remain below those of non-Indigenous populations globally (1, 2). Women from marginalised communities, including racial and ethnic minorities, often report higher rates of dissatisfaction due to systemic biases and discrimination in healthcare settings (3, 4). For many Indigenous women from remote areas, birthing in mainstream hospitals is the only available option (5–7). While for Indigenous women living in urban areas, birthing in mainstream hospitals is often a financial imperative driven by income (8).

In Australia, consequences of assimilation policies have resulted in poor socio-economic conditions combined with high rates of chronic diseases such as diabetes, which can lead to premature birth and the corresponding low birth weight and neonatal death (9). These factors also exist in countries like Canada, US, and Aotearoa (10, 11), and in Sápmi women’s birth outcomes have been inadequately addressed (12–14). These factors combined with culturally inappropriate health services can have an adverse impact on pregnancy outcomes (7). Dissatisfaction in the birthing experience, can erode trust in healthcare providers and systems, potentially deterring women from seeking necessary prenatal and postnatal care (15).

Existing research has reported on midwives’ and health care professionals’ perspectives on the care of Indigenous women (16, 17). However, knowing about the complex area of women’s dissatisfaction with birthing experiences is vital for improving healthcare quality, enhancing patient satisfaction, and ensuring better health outcomes for mothers and their children. Additionally, addressing the concerns of Indigenous women can enhance patient satisfaction, reduce postpartum mental health issues, and ensure safer, more positive outcomes for mothers and babies and is a matter of health equity (18). Systemic inequities impact Indigenous women’s health outcomes, underscoring the critical need for improvements in culturally safe healthcare practices and emphasises the need for a holistic, respectful approaches to maternity care that honours women’s voices and choices (3). This scoping review therefore aimed to identify studies reporting on what is known about Indigenous women’s dissatisfaction of birthing experiences in mainstream maternity hospitals in Australia (Aboriginal and Torres Strait Islander), Aotearoa (Māori), Canada (First Nations, Metis, and Inuit), US (Native Americans), Kalaallit Nunaat (Inuit) and Sápmi (Sámi) to identify gaps in existing literature and the need for future research.

This study uses the Indigenous names Aotearoa for New Zealand, Sápmi for the Sámi land areas and populations of Norway, Sweden and Finland, and Kalaallit Nunaat specifically because these terms are actively used by Indigenous communities within these regions and are increasingly recognised internationally. While Canada, the US, and Australia also have rich Indigenous histories, there are currently no widely accepted single Indigenous names for these entire countries. Our choice reflects the specific cultural preferences of the communities involved in this study and acknowledges their distinct identities.

In 2023, Indigenous births comprised 8.6% of Australia’s total births (24,737 Aboriginal and Torres Strait Islander births) (19), and Māori births represented over 30% of births in Aotearoa, despite the Māori population making up 17.1% of the national population (20). In Canada, Indigenous peoples (First Nations, Inuit, and Métis) account for 5% of the population, though Indigenous-specific birth numbers are unavailable for the 351,878 total births (21). In the US, 2022 saw 35,843 Indigenous births, accounting for 1% of the total (22), while Kalaallit Nunaat reported 716 births in 2023—the lowest birth rate since WWII (23). In Sápmi, estimates place the largest Sámi population on the Norwegian side of Sápmi at around 60,000, just over 1% of its total population, but there are no Sámi statistics and estimates are made based on geographical locations of inhabitants in the Sámi areas and municipalities (24). Indigenous populations in these regions face ongoing and complex historical, social, and health challenges due to impacts from colonisation, including land dispossession, cultural disruption, intergenerational trauma. Restricted access to healthcare in remote areas exacerbates these challenges, with systemic barriers and institutional racism contributing to poorer health outcomes. Nevertheless, Indigenous communities continue to champion self-determination, cultural revitalization, and environmental justice (19–21, 23, 25–27). Despite these obstacles, Indigenous communities are resilient, advocating for self-determination, cultural revitalisation, and environmental justice. The review focuses on birthing experiences while acknowledging the shared historical and social factors that influence health outcomes among Indigenous women across these countries.

This review was conducted in response to negative feedback from Indigenous women accessing perinatal healthcare within an Australian mainstream healthcare facility. To justify changes to the delivery of care and prior to conducting local research, it was important to have a thorough understanding of Indigenous women’s dissatisfaction in birthing in mainstream facilities globally. This review will advance the field by creating new knowledge of factors that influence Indigenous women’s birthing experiences, as well as inform evidence-based practice and improve the birthing experiences of Indigenous women globally.

## Methods

A scoping review was selected for its exploratory nature, as it systematically maps existing literature, helps understand current knowledge, and identifies key concepts and gaps (25, 28). A scoping review, unlike a systematic review, explores all available evidence on a broad topic. It is used when literature is diverse, complex, and requires broad exploration to identify gaps (28). A scoping review is particularly relevant when informing evidence-based healthcare and to incorporate knowledge into clinical practice (29). For this reason, a scoping review was selected as the most suitable form of review for this research. This review adhered to the framework established by Arksey and O’Malley (30) and incorporated recent methodological updates (28). The process involved five stages: (1) defining the research question, (2) identifying relevant studies, (3) selecting studies, (4) organising and charting the data, and (5) collating, summarising, and reporting the findings, as outlined by Arksey and O’Malley (30). The review was conducted based on a protocol that was registered in OSF^1^ prior to the study Sivertsen et al. (31). The reporting of this review adhered to the guidelines provided by the Preferred Reporting Items for Systematic reviews and Meta-analyses extension for Scoping Reviews (PRISMA-ScR) (32).

### Study population

The studies included in this review draw from women’s birthing experiences in mainstream maternity healthcare, which is medical facilities or highly specialised healthcare, mostly provided as a hospital in-patient on referral from primary or secondary health settings and can include complex medical or surgical procedures (62, 63). This review will focus on studies focussed on Indigenous women’s birthing experiences in Australia, Aotearoa (New Zealand), Canada, US, Kalaallit Nunaat (Greenland) and Sápmi, the cultural region traditionally inhabited by the Sámi people, assimilated by and spanning parts of countries Norway, Sweden, Finland, and to Russia’s Kola Peninsula. The Sámi are the Indigenous people inhabiting Sápmi, which is not a political entity, but a cultural and geographic area significant to the Sámi people. These countries comprise Indigenous populations, while also having similar histories inclusive of assimilation, and similar approaches to health systems and services (6, 33). All eight countries have diverse, multicultural populations, free and open presses and are closely aligned on key social and political issues (excluding the Russian part of Sápmi who is political and health systems, along with its lack of free press, differ vastly from those of Norway and other Scandinavian countries in Sápmi, leading to distinct challenges for the Sámi people in each region).

### Defining the research question

The research question guiding this review was: What is known about Indigenous women’s dissatisfaction of birthing experiences in mainstream maternity hospitals in Australia, Aotearoa, Canada, US, Kalaallit Nunaat and Sápmi? Including these countries in a scoping review on Indigenous women’s dissatisfaction with birthing in mainstream hospitals is justified due to the shared history of colonisation and the significant Indigenous populations in these countries. Each of these countries has distinct yet comparable healthcare systems and policies impacting Indigenous communities. By examining these countries, the review can provide a comprehensive understanding of how mainstream healthcare services address or fail to address the cultural needs of Indigenous women, offering insights into common challenges and potential solutions across different contexts.

For the purpose of this review, “dissatisfaction of birthing experience” was defined broadly to encompass any expressed discontent or unmet expectations regarding the cultural safety, accessibility, and quality of care received during the perinatal period in mainstream settings. The review goal was to gain a comprehensive understanding of factors contributing to Indigenous women’s dissatisfaction globally, providing a foundation for justifying changes in care delivery and guiding future local research on this issue.

### Identifying relevant studies

The search strategy aimed to locate both published and unpublished studies. A three-step search strategy was utilised in this review and considered primary research studies that report on reasons for dissatisfaction of birthing experiences, and strategies implemented to improve quality of clinical practice around women’s dissatisfaction of birthing experiences in mainstream maternity hospitals.

To ensure rigorous evidence synthesis, a research protocol was developed highlighting the inclusion and exclusion criteria and identifying how data would be extracted and presented. This was registered on the Open Science Framework (OSF) on May 14, 2024 (31).

A preliminary search was conducted in Medline (via Ovid) to identify articles, keywords and scope of topic. The identified keywords guided development of a comprehensive search strategy in Medline (see Supplementary material). Keywords included combinations of all variations of the following words: “Indigenous,” “birthing,” “dissatisfaction,” “hospital,” “mainstream.” The retrieved articles from the preliminary search were assessed to ensure the inclusion of key publications. The search strategy, including keywords and relevant index terms, was adapted for other bibliographic databases by a research librarian (JG), including PsycINFO (via OVID SP), Cumulated Index to Nursing and Allied Health Literature CINAHL (EBSCOhost), Web of Science (Clarivate Analytics), Scopus (Elsevier), ProQuest Central, ProQuest Social Science Premium Collection (Clarivate). ProQuest Dissertations and Theses was searched for unpublished material. Each database search strategy was run on May 28, 2024. Additionally, a supplemental search was conducted using Google Scholar and hand searching of key journals to identify studies meeting inclusion criteria. The search included articles from the inception of each database until 2024, with no restrictions on the publication date of the articles meeting the keyword inclusion criteria.

To expand the search, reference lists of all included sources were screened for additional studies. The 22 citations were also entered into Research Rabbit, an Artificial Intelligence (AI) tool for data mining scholarly publications, to conduct a final forward/backward search and identify timeline trends (34). This Artificial Intelligence (AI) tool is designed to support unstructured searching via data mining of publicly available scholarly papers and information relevant to seed papers uploaded (35). The tool was used for two main purposes: (1) to perform a final forward/backward check for any items that might have been missed during the systematic and hand-searching processes, and (2) to identify timeline trends for the included studies and related works. No additional relevant studies were found.

### Selecting studies

**Figure 1:**
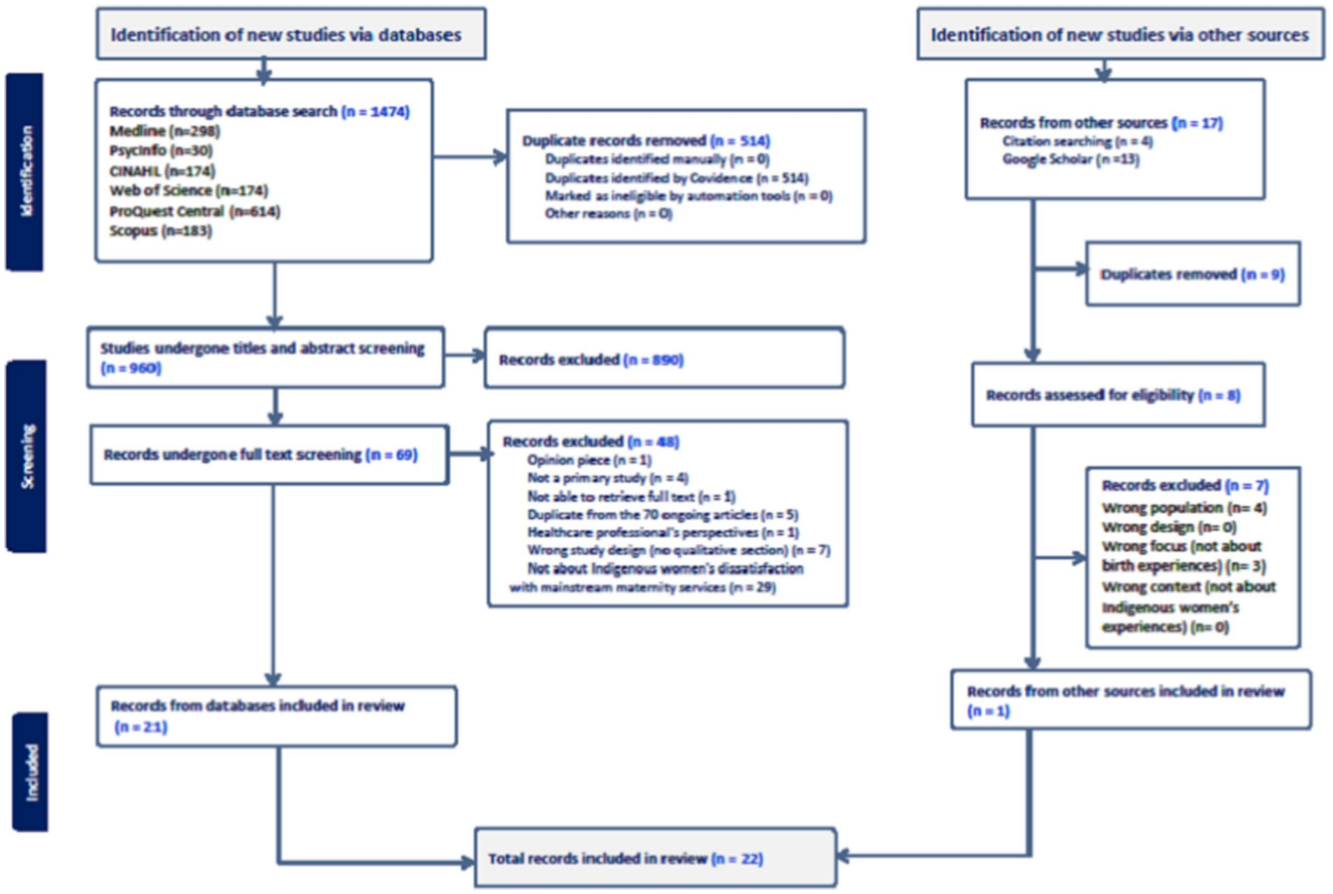
PRISMA flow chart (64).

For an illustration of the search and screening process see Figure 1 (PRISMA). The study titles and abstracts were screened for relevance, and if there was insufficient abstract information to determine eligibility the full text was retrieved. Full-text articles were evaluated against the following criteria: (a) those that included Indigenous women accessing perinatal services in mainstream health facilities; (b) those studies located in the geographical area of Australia, Aotearoa (New Zealand), Canada, Kalaallit Nunaat (Greenland), and Sápmi; (c) those which presented the results of peer-reviewed and non-peer-reviewed research based on qualitative or mixed-methods methodology that provided data on Indigenous women’s dissatisfaction in the perinatal in mainstream health facilities.

Language was restricted to English publications from inception of databases onwards. Studies were excluded if they (a) were an opinion article; (b) not a primary study; (c) not able to retrieve full text; (d) duplicate from the included full text articles; (e) no qualitative section of study; (f) not responding to the research question; or (g) studies published in languages other than English.

The inclusion and exclusion criteria were decided upon through discussions with the research team, which comprised three Indigenous and two non-Indigenous members. Both the perspectives of Indigenous women and of non-Indigenous women birthing Indigenous babies were included. By this, we refer to cases where a non-Indigenous mother and an Indigenous father have an Indigenous child. We included studies that presented firsthand accounts from Indigenous women or from non-Indigenous women birthing Indigenous babies, as well as studies documenting women’s experiences and implemented strategies to address dissatisfaction in care. We excluded studies that did not contain Indigenous women’s voices or direct experiences, including those focused on healthcare professional perspectives, as these may not accurately represent Indigenous women’s experiences. Additionally, studies reporting general birthing stories, which do not specifically address Indigenous women’s unique experiences and concerns, were excluded. This approach ensured that our review centred on the firsthand perspectives of Indigenous women regarding perinatal care.

Following the search, the 1,474 citations identified were collated and uploaded into Covidence (36), and 514 duplicates removed. After pilot testing, 960 articles underwent title and abstract screening by four reviewers (NS, TMN, GM, and TJ). The relevant studies were retrieved in full, and their citation details imported into Covidence. The full text of 69 citations was assessed in detail against the inclusion criteria by four independent reviewers (NS, GM, SS, and TJ). A manual search in Google Scholar and thorough examination of the reference lists of the included articles retrieved a further 8 articles which were also screened for eligibility. The reasons for excluding papers at the full-text stage, which did not meet the inclusion criteria, were documented as shown in Figure 1 (PRISMA chart). Disagreements between reviewers during the selection process were resolved through discussion or, if necessary, with the involvement of an additional reviewer. There were seven conflicts in Covidence, attributed to ambiguity around what mainstream health services encompass. These disagreements were resolved through discussion among the reviewers to ensure consistency and rigour in our inclusion decisions. This collaborative approach helped us reach a consensus on all disputed studies, strengthening the reliability of our selection process.

At the end of the selection process, 22 full-text articles were identified.

### Organising and charting the data

Three reviewers (NS, TMN, and SS) independently evaluated each article that met the inclusion criteria using a data extraction tool created by the authors, adapted from the JBI extraction tool (37), which was pilot tested and then used in Covidence for extraction. The reviewers (NS, TMN, and SS) extracted details such as author names, publication date, title, study setting, study design, data collection methods, and sample characteristics. Also extracted were outcome of studies in relation to dissatisfaction of birthing in mainstream hospitals and critical findings.

Any disagreements during the data extraction process were resolved through discussion between the reviewers to reach consensus. Four reviewers (NS, SS, TMN and TJ) collaboratively identified key themes and patterns to provide a descriptive overview of the main content and findings of each article, in line with the review’s objective. These themes and patterns were then discussed, further developed, and refined in consultation with the entire authorship team.

### Collating, summarising, and reporting the findings

The included articles were analysed using basic descriptive summaries following a template developed and pilot tested by two authors (NS and SS). This abridged thematic synthesis (38) approach to assess and extract data from the included studies involved summarising content across studies and identifying recurring themes related to Indigenous women’s dissatisfaction with perinatal care. Key parameters guiding the analysis included cultural safety, accessibility of services, continuity of care, and experiences of discrimination within healthcare settings. This adapted thematic synthesis (38) allowed us to systematically organise and interpret findings, providing a comprehensive view of Indigenous women’s perinatal care experiences in mainstream settings. This analysis was guided by the research question of the scoping review.

**Figure 2:**
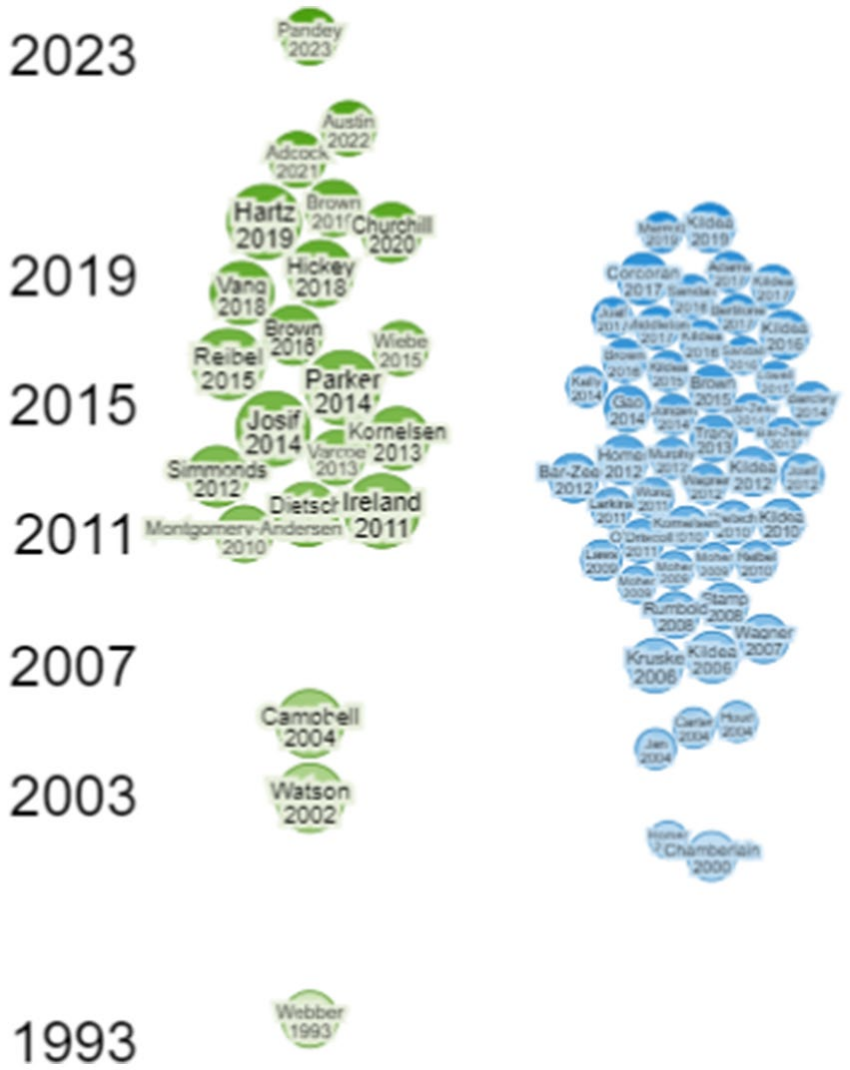
Timeline of selected papers and similar works.

The Research Rabbit AI tool generated a timeline visualisation for the 22 studies (shown in green) and identified 679 similar works, of which the 50 most relevant works (shown in blue) are presented in Figure 2. The visualisation highlighted growing interest in Indigenous women’s birthing experiences from 2011 to 2021. However, the similar works primarily focused on health care professional’s perspectives of providing care to Indigenous women and families, community service provision, antenatal services in remote locations, and models of midwifery care.

## Results

### Key characteristics of included studies

Database searches resulted in 1,474 initial records, then 514 duplicate records removed, with 890 excluded following title and abstract screening. We assessed 69 full texts for eligibility, 48 were excluded. One study was included from Google Scholar. This meant that *n* = 22 studies, published within the last 21 years (1993–2024), met all eligibility criteria, with a total of 1,578 participants. Key characteristics of the included studies are summarised in Supplementary Table 1 where each study is numbered and referred to in the results reported here.

### Participants

Study sizes ranged from 4 to 344 participants, with an average of 72. Together, the studies included 1,437 women, 36 Elders, 14 fathers and family members, and 91 healthcare professionals.

#### Setting

The studies were conducted across eight countries. The majority were conducted in Australia (*n* = 11), with 9 in Canada, 1 in Aotearoa, and 1 in Kalaallit Nunaat.

#### Overall study quality

Although the quality of evidence was not assessed using a quality assessment tool, as the goal of this scoping review was to provide an overview of the existing evidence, irrespective of quality (28), the reviewers conducted a structured review of each study’s credibility, dependability, confirmability, transferability, and authenticity. This assessment involved examining each study’s methodology for rigour, sample size for representativeness, and limitations for transparency. Studies were evaluated on their design strength, relevance to Indigenous women’s experiences, and the reliability of their findings. Based on this approach, we categorised studies broadly by quality, noting that while some studies had smaller samples or limited methodological detail, they still offered valuable insights. This approach ensured that, while prioritising inclusivity due to the scarcity of studies, a rigorous analytical lens was still applied to interpret and contextualise the findings appropriately.

Additionally, the experiences of Indigenous women represented at least 11 specific cultural groups including Australian Aboriginal women from Ngaanyatjarra, Aboriginal and Torres Strait Islander women from Western Australia, Queensland, the Northern Territory, New South Wales, and Victoria (Cultural groups not identified), Canadian Inuit women and Aboriginal women from Alexander, Alexis, Enoch, Paul, Nuxálk, Haida and Namgis nations. Indigenous women from Wemindji Cree nation, Cree from Moose Factory zone and Stoney nation. Finally, Māori women from Aotearoa as well as Inuit women from Kalaallit Nunaat. The reviewed articles focused on countries with Indigenous populations, but most studies located were from Australia and Canada with one each from Aotearoa, and Kalaallit Nunaat.

The 22 documents selected, listed in Supplementary Table 1, are a combination of research articles and theses. These highlighted the similarities of experiences of Indigenous women who used perinatal care in mainstream hospitals in regional centres in Australia, Aotearoa, Canada, US, and Kalaallit Nunaat. The reviewed articles focused on countries with Indigenous populations, but most studies located were from Australia, Canada, Aotearoa, and Kalaallit Nunaat. The findings from the studies identified similar trends across the studies. These included the strong preference by Indigenous women to birth in their local community; a desire for the inclusion of support person in the evacuation process; a preference for continuity of care; a need for cultural safety education of staff as well as a preference for female health care providers. These studies also revealed that evacuation to regional hospitals resulted in a lack of social support, feelings of loneliness and isolation (Table 1).

**Table 1:** Critical findings.

Critical findings of the review
The narratives reveal the necessity of integrating critical medical anthropology and cultural safety perspectives into health care to address deeply ingrained issues, as health care encounters reflect the social, political, economic, and ideological relations between patients and the dominant health care system.
Childbirth evacuation is a stressful and isolating experience for Indigenous women who showed a strong preference for a Birthing on Country model of care and having a support person present.
Cultural safety issues were identified along with fragmented care and some staff who did not listen and were unsupportive. This highlighted a need for cultural safety training of all staff and greater presence of Aboriginal support staff.
Aboriginal community-controlled health services are well placed to provide appropriate and accessible care to Indigenous women during pregnancy and the postnatal period.
Continuity of care including through a Midwifery group practice was well received by Aboriginal women.
Prior negative experiences impacted women seeking pre-natal care.
Strong need to prioritise cultural needs in birthing hospitals.

### Preference for community based antenatal and perinatal care and the effects of evacuation on indigenous women

While many Indigenous women are aware of the dangers inherent in birthing without appropriate supports, all studies identified a preference for birthing in home communities or as near as possible, if appropriate services were available (10, 33, 39–41). The Australian study by Ireland et al. (5) identified that the current model of care which involved evacuation to regional hospital, as an infringement of Women’s Business laws. The participants in the study believed that all matters relating to reproduction are “Women’s Business,” and while the boundaries of Women’s Business are changing, this study highlighted the dislocation of birthing women from their culturally appointed carers. The women reporting ongoing emotional impacts, and lasting emotional impacts of shame and technological violation during childbirth. This preference to receive antenatal and perinatal care in the community was also evident in Canadian and Māori Indigenous women (40, 42). Indigenous women universally reported that evacuation to a regional hospital resulted in a geographic and emotional disconnect from community, a lack of social supports and feelings of isolation, lack of meaningfulness and concern about the wellbeing of older children left behind in communities (6, 7, 10, 43–45). The Canadian study by Wiebe et al. (7) further highlighted how according to elders, the separation of childbirth from the geographic and cultural community, from the woman’s home community, is traumatic, and this trauma also changed the way childbirth was experienced by labouring mothers. In addition to the isolation, mothers also reported greater physical pain when birthing in the hospital, compared to at home or in their community. In Canada, Kornelsen (40) study found that women reported feeling powerless in the urban birthing environment, and missing family, and estrangement from cultural norms.

### Importance of culturally appropriate support person

Many studies identified the need for a culturally appropriate support person for women who are evacuated to regional hospitals while awaiting the birth of their child (40–42, 46, 47). The studies highlighted the value family support to improve women’s experiences of care (41). It was identified that evacuation to a regional hospital results in the loss of emotional support from their family members and culturally appointed carers (5, 40, 42). Additionally, the study by Ireland et al. (5) identified that a support person can assist with appropriate communication, can act as a cultural adviser, and can enhance wellbeing and improve experiences of care (41, 42, 47). Women in a Canadian study (48) emphasised that giving birth alone and being separated from family could negatively impact their mental health, whereas being accompanied by a partner could support their overall wellbeing. Additionally, the adoption of a support person was found to prevent misunderstandings in discussions with healthcare providers and may also improve attendance at antenatal care (47). There is evidence to suggest that there is a strong cultural connection for the family when a baby is due and without support the complication of relocation to a regional acute care facility may result in resistance to attend antenatal classes and disengagement from antenatal care in general (46). The inclusion of Aboriginal health workers and greater numbers of Aboriginal midwives may improve the women’s’ feeling of safety and being welcomed in an unfamiliar place (46).

### Benefits of continuity of care

Three of the studies included in this scoping review evaluated the perceptions of Indigenous Women who attended venues employing continuity of care practices (17, 41, 49). All studies reported positive experiences from participating in this type of care and participants expressed appreciation of having a health care provider with knowledge of their background and insight into living within a remote environment (17). Aside from enhanced continuity of care, one study reported greater cultural responsiveness of the wider midwifery services and staff (17). The women reported feeling culturally safe, connected, and supported while experiencing emotional and clinical safety (49). A further positive finding was the presence of respectful relationships and shared decision-making (49). However, not all findings were positive, and negative experiences persisted including structural and systemic issues such as fragmented care and unsupported or culturally unsafe care within the broader health system (49). While the continuity of care model is safe and highly valued by Indigenous women, there was evidence to suggest that some women still experienced incidences of being ignored, having concerns dismissed, experienced racism and felt talked down to and limited choice in care (41).

### Lack of cultural understanding, empathy and presence of discrimination and racism in healthcare

Lack of cultural understanding or relationships and trust were pervasive in many studies, and Brown et al. (50) Australian study found up to 51% of women reported receiving discrimination or unfair treatment from hospitals or health services during pregnancy or shortly after. Vang et al. (44) study in Canada, found issues of perceived cultural stereotypes, labelling, communication issues and medical mistrust were identified. Women identified hospital bureaucracy including waiting times, the feeling of being rushed and lack of time to build relationships with staff as a negative experience creating stress. The Australian study by Dietsch et al. (39) reported that women are impacted by closures of maternity services in rural areas, and experience feelings of loneliness, fear, isolation, and alienation when forced to relocate to large city hospitals to deliver. They felt disrespected, oppressed, culturally unsafe and experienced racism. The study recommends that Aboriginal women’s’ ties to country (Birthing on Country) and kinship networks should be valued and models of midwifery care developed to ensure healthy pregnant women have a choice as to whether they remain on country or transfer, from their country and kin, to birth (39).

Watson (45) study in Australia found women experienced feelings of loneliness when confined to birth in a mainstream hospital. Participants reported inadequate interactions with staff, were frightened and described miscommunication, lack of empathy and misunderstanding of cultural and spiritual beliefs. Additionally, the women described negative experiences including the need for explanations regarding their birthing experience. This was also found in the Canadian study by Varcoe et al. (10) where most participants described distressing experiences during pregnancy and birthing including healthcare professionals who lacked understanding of historical and ongoing colonial relations which impacted choice and affect birth outcomes. The critical finding of this study was that prior negative experiences and discrimination were deterrents to accessing prenatal care.

It has become evident throughout this review, that healthcare professionals can have a profound effect on women’s perception of the birth experience and encounters with healthcare professionals can be positive or negative (10, 44). The studies included in this review cited incidences of judgment, racism, cultural misunderstandings, poor relationships, improper care, patronising behaviour as well as demeaning and poor communication (10, 19, 33). Health inequities are evident, and in Australia Aboriginal women are most at risk of poor infant health outcomes and they are also the least likely to perceive that they received care well matched to their needs (50).

### The need for cultural safety education of healthcare providers

Indigenous women, most at risk of poor infant health outcomes, were the least likely to perceive that the care they received was well matched to their needs (50). Many of the studies included in this review identified the need for cultural safety education of health care providers as well as greater inclusion of Indigenous perspectives (7, 10, 33, 43, 44, 46, 48, 49, 51–53).

A Canadian study Vang et al. (44) highlighted the social disconnect and social isolation experienced and concluded the need for institutions to instigate cultural sensitivity training that highlights the larger historical, social, and political issues experienced by Indigenous women. Women in Varcoe (10) study described distressing experiences during pregnancy and birthing including healthcare professionals who lacked understanding of historical and ongoing colonial relations which impacted choice and affect birth outcomes. Churchill et al. (52) found that culturally safe care included having personalised continuous relationships with midwives, and having a space that made participants feel “at home” and acknowledged that Indigenous and non-Indigenous participants conceptualised and experienced cultural safety in diverse ways (52). In Australia, women reported feelings of being judged by midwives and that this negatively impacted their experience (43). However, the same study reported that positive outcomes came when staff practised open inclusive communication with the women (43). The critical finding of this study is that culturally safe care, emphasising inclusive communication and active decision-making involvement, is essential to improve childbirth experiences for Aboriginal and Torres Strait Islander women, who often face judgment and cultural misunderstandings (43). Similarly, the Canadian study by Browne and Fiske (51) found a need for cultural safety training to ensure Western nurses challenge their own cultural assumptions about Indigenous women.

## Discussion

This scoping review has identified the need for culturally safe trauma informed care, inclusive communication, active decision-making involvement and greater inclusion of Indigenous perspectives including the involvement of Indigenous birth support workers where appropriate (7, 10, 33, 43, 44, 46, 51–53). It is time that mainstream healthcare systems and services acknowledge the impact of majority perspectives in healthcare and the impact this has on Indigenous families. Indigenous women live the consequences of colonisation every day. The medicalisation and hospitalisation of childbirth has resulted in the loss of birthing knowledge in communities, and women report experiencing limited cultural safety in hospital and evacuation settings. Some may argue that the term transfer or referral should replace the word evacuation, however evacuation is widely used in the literature that emerged in this review, and it is retained to accurately reflect the lack of choice that many Indigenous women face when needing to leave their communities for birthing. Unlike referral or transfer, which imply a degree of agency or choice, evacuation conveys the reality that many Indigenous women are required to leave their local communities, often under urgent circumstances, with little or no input in the decision. This term captures the imposed nature of these relocations and aligns with the language commonly employed in studies addressing similar contexts. Recommendations include the urgent need to reintegrate culturally based community support and health perspectives into the childbirth experience (7).

There was a strong preference for a Birthing on Country model of care where possible (10, 33, 39, 40, 47). A Birthing on Country model of care is a culturally safe, community-focused maternity care approach for Aboriginal and Torres Strait Islander women that integrates traditional practices and supports holistic wellbeing. Cousins (54) highlights the inequities faced by Indigenous mothers and babies in Australia, where they experience significantly worse pregnancy outcomes compared to non-Indigenous populations. These disparities are driven by a combination of limited access to healthcare in rural and remote areas, cultural disconnection, and broader social determinants such as poverty and housing overcrowding, because of colonisation. The Birthing on Country movement aims to restore culturally safe, community-based birthing services under Indigenous control, emphasising the importance of traditional practices, connection to land, and holistic care. Studies show that models incorporating continuity of midwifery care, cultural safety, and Indigenous-led services have led to improvements in birth outcomes, including reduced preterm births and better maternal and infant health (3, 33, 55). Campbell and Brown’s (56) study, suggest that Aboriginal Community Controlled Health Services are well placed to provide appropriate and accessible care to Indigenous women during pregnancy and the postnatal period. However, despite the success of these innovation of services and initiatives, researchers are calling for broader government investment to expand a Birthing on Country model of care across Australia to further address these systemic inequities (54). This investment should also include ways to eradicate discrimination and racism in health care.

The findings of this review described distressing experiences, racism and discrimination during pregnancy and birthing including healthcare professionals who lacked understanding of historical and ongoing colonial relations which impacted choice and affect birth outcomes (10, 33, 39, 50). There is a strong need to eliminate discrimination and racism in maternity care by addressing the intersectional power dynamics that shape maternal health outcomes. It underscores how structural inequalities, including racism and gender oppression, harm the care of marginalised groups, especially women of colour and Indigenous women. To combat these inequities, research calls for culturally safe, person-centred care and urges healthcare systems to adopt an intersectional approach in policy and practice design to promote maternal health equity (15, 57).

This scoping review included Sápmi from the onset, however we were not able to identify research studies exploring dissatisfaction around Sámi birthing experiences. Finding zero articles about Sámi dissatisfaction with birthing experiences in a scoping review could indicate a significant gap in the existing research and scholarship on this topic. A Sámi book about birth stories reveals both positive and negative experiences from giving birth. Several stories in the book describe how Sámi women experience a lack of cultural understanding and accommodation in maternity care, leading to feelings of insecurity, isolation, and frustration. The book emphasises the importance of culturally sensitive care and a healthcare system that more respectfully and inclusively addresses Sámi women’s needs (58). Compared to other experiences reported by Sámi patients, encounters with public healthcare often reveal that healthcare professionals neglect key aspects of the patient’s cultural identity. These include the patients’ sense of being in a culturally unsafe environment and their feelings of disconnection from their land, culture, language, and family (59, 60). The general recommendations from WHO (11) is that childbirth should be evidence-based, family-centred and involving the mother in decision-making processes. Additionally, they stress that perinatal care should be assessed and adjusted into a cultural safe practice. Whether this is the case for Sámi women remains to be studied. Despite the significant presence of Sámi communities across Sápmi, this notable lack of research on Sámi women’s birthing experiences in mainstream hospitals overlooks the unique cultural needs and challenges faced by Sámi women during childbirth, particularly in navigating a healthcare system that may not fully understand or accommodate Sámi cultural practices and values. This absence suggests that the specific concerns and experiences of Sámi women in the context of childbirth have been overlooked or insufficiently explored in academic and healthcare studies, and that the specific concerns of Sámi women—such as the importance of language, traditional practices, and the potential for cultural disconnect—remain underexplored. It highlights the potential invisibility of Sámi voices in discussions about healthcare equity and cultural safety, underscoring the need for targeted research to better understand and address the unique needs of Sámi women in maternity care. Addressing this research gap is crucial to ensuring culturally safe and respectful care for Sámi women in the Norwegian, Swedish and Finnish healthcare system.

It should be noted that countries in Sápmi, such as Norway, do not statistically collect data on who is Sámi (61), which presents significant challenges in understanding and addressing the specific needs of the Sámi population. The absence of such data limits the ability to identify and analyse disparities in health, education, and socio-economic status between the Sámi and the broader Norwegian population. Without statistical recognition, the Sámi people remain largely invisible in national data sets, making it difficult to develop targeted policies or interventions that reflect their unique cultural and social circumstances. This lack of data collection also complicates efforts to monitor and ensure the protection of Sámi rights, contributing to the marginalisation of Sámi communities within Norway.

### Limitations

This is the first review to systematically synthesise existing evidence on Indigenous women’s dissatisfaction of birthing in mainstream hospitals. The strength of this scoping review is its novel nature. Additionally, a further strength is in its global approach to the experiences of Indigenous women. This review has included the experiences of Indigenous women across eight countries and at least 11 specific cultural groups including Australian Aboriginal women from Ngaanyatjarra, Aboriginal women from Western Australia, Queensland, the Northern Territory, New South Wales and Victoria (Cultural groups not identified), Canadian Inuit women and Aboriginal women from Alexander, Alexis, Enoch, Paul, Nuxálk, Haida and Namgis nations. Indigenous women from Wemindji Cree nation, Cree from Moose Factory zone and Stoney nation. Finally, Māori women from Aotearoa as well as Inuit women from Kalaallit Nunaat. However, the findings should be considered with acknowledgement of the following limitations. The review included only English-language studies, potentially missing relevant studies in other languages. Although the search strategy was comprehensive, studies around dissatisfaction of Indigenous women’s birthing published in non-traditional mediums or grey literature may not be fully represented. The generalisability of the results to non-English-speaking contexts is uncertain, and future research could expand to include non-English studies. While grey literature was not excluded from the initial search no unpublished papers were detected.

## Conclusion

This review has confirmed that the medicalisation and evacuation of Indigenous women for childbirth has resulted in a cultural and social disconnect for many women. While many women are aware of and appreciate the improved safety for themselves and their infant, the studies show a preference for a Birthing on Country model of care where possible, and a strong preference for receiving antenatal care in the community. The findings also showed that the care provided to evacuees is largely suboptimal with many women reporting feelings of boredom, isolation, and loneliness. However, it has become evident that the continuity of care experience has enhanced the experience of Indigenous women. Despite this, many studies identified a lack of cultural safety present in the mainstream hospital environments. These findings point to a strong need for cultural safety education of health care providers and greater emphasis being placed on communication, empathy, and the incorporation of cultural and spiritual practices in the hospital environment. Finally, the importance of a support person or Indigenous Birth Support Worker was shown to have benefits both antenatally and in some cases, perinatally.

## Footnotes

^1^ https://doi.org/10.17605/OSF.IO/URYFG
