# Supplementary material for: What is known about Indigenous women’s dissatisfaction of Birthing experiences in mainstream maternity hospitals in Australia, Aotearoa, Canada, US, Kalaallit Nunaat and Sápmi? A systematic scoping review
Source: Front Public Health. 2025 Mar 21;13:1495197. doi: 10.3389/fpubh.2025.1495197 (PMC11970129; doi:10.3389/fpubh.2025.1495197)
Supplement: Supplementary file 1 [file Data_Sheet_1.pdf]

ADDITIONAL MATERIAL 1

Full Medline search terms:

#	Searches	Results
1	indigenous peoples/ or "american indian or alaska native"/ or indians, north american/ or alaskan natives/ or indigenous canadians/ or inuit/ or navajo people/ or pima people/ or "australian aboriginal and torres strait islander peoples"/ or maori people/ or "native hawaiian or other pacific islander"/	33275
2	(Aboriginal* or Torres Strait Islander or ATSI or (Australia* adj3 (first nation* or indigenous or first people*))).tw,kf.	13070
3	((Austra* or New Zealand or Aotearoa* or Pacific island* or Hawaii*) adj3 (race* or native*)).tw,kf.	4125
4	(Kaurna or Adnyamathanha or Mula or Maralinga or Narungga or Ngaanyatjarra or Ngarrindjeri or Pitjantjatjara or Yolngu or Anangu or Yankunytjatjara or Arrernte or Aranda or Arunta or Arrarnta).tw,kf.	218
5	("A' ani" or Absaroka or Haaninin or Atsina or "Gros Ventre" or Acopsel or Tlacopsel or Lacopsel or Ahtna or Ahtena or Akenitsi or Occaneechi or Akokisa or Horcoquisa or Orcoquizas or Aleut or Unangax or Unangan or Alibamu or "Alabama Alsea" or Alutiiq or Sugpiag or Amahami or Awaxawi or Androscoggin or Arosaguntacook or Ameriscoggin or Anishinaabeg or Chippewa or Anihsinape or Saulteaux or Apalachee or Aranama or "Texan Coahuilteca" or Tamique or Arikara or Sahnish or Arickaree or Adakadaho or Assiniboine or Hohe or Nakota or Nakoda or Nakona or "Atsa' Kudok-wa" or Awatixa or Bannock or "Snake Indian*" or Bidai or Quasmigdo or Biloxi or Blackfoot or Niitsitapi or Sikasikaitsitapi or Cahto or Kaipomo or Cahuilla or Ivilyuqaletem or Ivilyuat or Catawba or Inna or Iswa or Chemehuevi or Chickasaw or "Chilula Chimakum" or Aqokulo or Chimariko or Chiricahua or Tsokanende or Chitimacha or Chetimachan or Sitimacha or Chowanoke or Roanoke or Chumash or Ciboney or "Taino Ciwat" or Clatsop or Coos or Coosa or Uchis or Chiaha or Coste or Talisi or Coquille or Kokwell or Coso or Cowlitz or Taitnapam or "Crow Nation" or "Cui Ui Ticutta" or Cupeno or Kuupangaxwichem or Cupa or "Cup' ig" or Eskimo* or Whenua* or Iwi* or Nunivak or "Dakota Oyate" or Lakota or Nakota or Santee or Teton or Sioux or Deadose or "Deg Xina" or "Deg Xit' an" or Kaiyuhkhotana or "Deg Hit' an" or "Dena' ina" or Tanaina or "Dichinanek' Hwt' ana" or "Upper Kuskokwim Athabascan*" or Kolchan or Goltsan or "Tundra Kolosh" or "Do lkabya" or Duwamish or Esselen or Eyak or "Gidi' tikadi" or Guwevkabaya or "Gwich' in" or Kutchin or Haida or Xaadas or Xaat or Halchidhoma or Havasupai or "Green Water People" or Hiratsa or Hiraaca or "Ho-chaaqa" or Winnebago or Holikachuk or Innoko or "Tlegon-khotana" or Hopi or "Houma-Louisiana" or Huaco or Waco or Hualapai or Hupa or Natinixwe or "Natinook-wa" or "Hwech' in" or Hankutchin or "Iroquois Confederacy" or "Hodinoso ni" or "Illinois Confedera*" or Ilinoweg or Illini or Inupiat or Inuit or Ioway or Baxoje or Jicarilla or Juaneno or Acjachemen or Jumano or Kalapuya or Kalaallit* or Clackama or Kalispel or "Pend d' Oreilles" or Qlispe or Karuk or Karok or "Chum-ne" or Katkoc or Kansa or Kanza or Kawaiisu or Nuwa or	17277

	Kennebec or "Kinipekw Kittitas" or Klickitat or "Qwu' lh-hwai-pum" or "Awi-adshi" or Mahane or Wahnookt or "Koa' aga' itoka" or Keresan or Kichai or Kitsai or Keechi or "K' itaish" or Kiowa or Gaigwu or Cauigu or Kutjau or "Kwu-da" or "Tep-da" or Kitanemuk or Kittitas or Klickitat or "Qwu' lh-hwai-pum" or "Awi-adshi" or Mahane or Wahnookt or "Koa' aga' itoka" or Konkow or "Koop Ticutta" or Koyukon or Ktunaxa or Kootenai or Flathead or Kucadikadi or "Kotsa' va" or Kumeyaay or "Tipai-Ipai" or Kamia or Diegueno or Kwapa or Cocopah or Cucapa or "Xawitt kwnchawaay" or Lassik or Lenape or "Leni-Lenape" or Lipan or Luiseno or Payomkawichum or Madqwadabaya or "Desert Yavapai" or Mahican or Mohicans or Makah or Makuhadokado or Maliseet or Wolistoqiag or Manahoac or Mahock or Meipontsky or Mandan or Mattole or "Bear River" or "Tul' bush" or "Ni' ekeni" or Meherrin or Menominee or Mackinac or Metis* or Mescalero or Myaamiaki or Kickapoo or Twigtwee or Missouria or Miwok or Miwuk or Moadokado or Modoc or Mohave or "Aha Makhav" or Mohawk or "Kaneng' hega" or Molala or Molale or Molele or Nyyhmy or Moosonee or "Moose Cree" or Monsonis or Multnomah or Chinook or Nabedache or Nabaydacu or Wawadishe or Nabiltse or Dakubetede or "Nacho Nyak Dun" or Tutchone or Nacono or "Na' isha" or Nanticoke or Navajo or Ndee or Nial or Niimiipu or "Nez Perce" or Watapala or Watapahlute or Nisenan or Nisqually or Nomlaki or Noamlakee or "Central Wintun" or Nongatl or Nottoway or Cheroenhaka or "Northern Cheyenne" or Ohlone or Costanoan or Omaha or "O' odham" or Pima or Papago or Osage or Otoe or Otse or "Ozav Dika" or Palus or Passamaquoddy or Pestomuhkati or Patiri or Petaros or Pastia or Patwin or "Southern Wintun" or Panis or Skidi or Pedee or Penobscot or "Petun Piipaash" or "Kokmalik' op" or Piscatawa or Doeg or Conoy or "Pit River" or Pomo or Kashaya or Ponca or Ponka or Pottawatomi or Bodewadmik or Powhatan or Puyallup or Spuyalepabs or Quapaw or Ugahxpa or Quechan or Yuma or Kwtsaan or Quileute or Salinan or Saponi or Monacan or Sapon or "Eastern Blackfoot" or Christanna or Sawawatodo or Serrano or Taaqtam or "Maarenga' yam" or Yuhaviatam or Shasta or Chasta or Sasti or Shoshone or Siletz or Sinkine or Sinkyone or "Siuslaw Umpqua" or Skitswish or "Schitsu' umash" or Snohomish or Snuqualmi or Sokoki or Missiquoi or Stillaguamish or Stoluckwamish or Suquamish or Sutaio or Swinomish or Skagit or Syilx or Okanagan or Sotaae or "Taga Ticutta" or Takelma or Dagelma or Taltushtuntede or Galice or "Tanan Gwich' in" or Taos or Taovaya or Tataviam or Alliklik or Tawakoni or Tahuacano or Tenino or Thawikila or Hathawekela or "Fort Ancient" or Tigua or Tillamook or Nehalem or Timbisha or Panamint or Timpanogos or Tlingit or "Toi Ticutta" or Tolowa or "Talawa Dini' " or Tongva or Gabrieleno or Fernandeno or Tobikhar or Tonkawa or Ticanwatic or Tsikip or Appalousa or Opelousa or Tsitsistas or Tubatulabal or Tukabatchee or Tuscarora or Tomahittan or Kuskarawock or Tutelo or Tutero or Totteroy or Tutera or Yusan or Tututni or Umatilla or Umpqua or Waccamaw or Waxmaw or Wadatika or "Harney Valley Paiute" or Wailiki or Waluulapam or "Walla Walla" or Walpapi or Huipui or Wampanoag or Massasoit or Wanapum or Wappo or Washoe or Wichita or Willapa or Kwalhioqua or "Wi pukba" or "Verde Valley Yavapai" or Wintu or "Northern Wintun" or Wiyot or "Wee' at" or Weyet or Yakama or "Yamosopo Tuviwarai" or Yaqui or Yoeme or Yatasi or Yattasih or "Yavbe' " or "Yavapai" or "Ysleta del Sur" or Yojuane or Yokuts	

	or Mariposa or Yuki or Yupighyt or "Yup'ik" or Yupik or Yurok or "Olekwo'l" or Zuni).tw,kf.	
6	((American or Canad*) adj3 (race or native or amerind* or Indian*)).tw,kf.	19111
7	((Applegate or Delaware or Iowa or Ishak or Kaw or Kato or Spokane or Miami or Arkansas or Tali or Tunica or Han or Pawnee or "Coeur D' Alene" or Piscataway or Ree or Tula) adj3 (reservation* or nation or people or peoples or population or man or men or woman or women or child* or youth* or elder or elders or communit* or tribe or tribes or tribal or Indian*)).tw,kf.	11906
8	(Maori* or Maori).tw,kf.	4641
9	(Lapps or Llaplander or Laplander* or Mapuche or M?ori or M?tis or Nava?o or Nunangat or Ojibwe or Sámi or Saami or Skolt or Taiga or "tangata whenua" or Wampanoag or Yuit or Yupik).tw,kf.	19834
10	Health Services, Indigenous/	4285
11	or/1-10	93638
12	exp Pregnancy/ or Postpartum Period/ or exp Parturition/ or Labor, Induced/ or Labor, Obstetric/ or Labor Pain/ or Labor Presentation/ or Labor Stage, First/ or Obstetric Labor Complications/	1038673
13	(pregnan* or postpartum or parturition or labor or childbirth or birth* or postnatal or intrapartum or postnatal or puerperium).tw,kf.	1116846
14	12 or 13	1532248
15	exp Hospitals/ or Birthing Centers/ or Academic Medical Centers/ or Delivery Rooms/ or Nurseries, Hospital/ or Hospital Units/ or Hospitalization/	480172
16	Obstetrics/ or "Obstetrics and Gynecology Department, Hospital"/ or Maternal Health Services/	43139
17	(hospital* or ward or Obstetrics or Gynecology).tw,kf.	1725087
18	or/15-17	1915092
19	((("semi-structured" or semistructured or unstructured or informal or "in-depth" or indepth or "face-to-face" or structured or guide or guides) and (interview* or discussion* or questionnaire*)) or experience* or perception* or perciev* or satisfact* or disatisfact* or "focus group" or "focus groups" or qualitative or ethnograph*OR fieldwork or "field work" or "key informant").tw,kf.	2361236
20	exp Qualitative Research/ or "Interviews as Topic"/ or Focus Groups/ or Narration/ or "Personal Narratives as Topic"/	170843
21	19 or 20	2398287
22	11 and 14 and 18 and 21	298
