# Supplementary material for: What is known about Indigenous women’s dissatisfaction of Birthing experiences in mainstream maternity hospitals in Australia, Aotearoa, Canada, US, Kalaallit Nunaat and Sápmi? A systematic scoping review
Source: Front Public Health. 2025 Mar 21;13:1495197. doi: 10.3389/fpubh.2025.1495197 (PMC11970129; doi:10.3389/fpubh.2025.1495197)
Supplement: Supplementary file 2 [file Table_1.docx]

Table 1 Summary of included articles

| **Covidence #** | **Study ID** | **Title** | **Design  Methods Participants** | **Country and Culture Group** | **Aims and objectives of the studies** | **Indigenous research methods for data collection and analysis** | **Outcomes** | **Critical finding** |
| --- | --- | --- | --- | --- | --- | --- | --- | --- |
| 1400 | Wiebe et al., 2015. | Restoring the Blessings of the Morning Star: Childbirth and Maternal-Infant Health for Indigenous near Edmonton, Alberta. | Participatory action research using a descriptive qualitative design.  75 participants. | Edmonton, Canada, Aboriginal and Indigenous Women. | To explore childbirth experiences of Indigenous women. | Talking circles (seven) were employed. n=75 Cree, Stoney Alexis, Enoch and Paul Indigenous women participated in the talking circles. Five semi-structured interviews were also conducted with four respected traditional elders. Stories told by the participants were appreciated in their entirety during the analysis and  thus coded in their entirety to respect the Aboriginal form of transferring  knowledge through storytelling. Data were managed manually and coded. | The medicalisation and hospitalisation of childbirth has resulted in the loss of birthing knowledge in communities. Participants reported limited cultural safety in hospital setting.  Recommendations include the urgent need to reintegrate culturally based community support and health perspectives into the childbirth experience. | According to the Elders, the separation of childbirth from the geographic and cultural  community, from the woman’s home community, is traumatic, and this trauma changed the way childbirth was experienced by labouring mothers. In addition to the isolation mothers  felt, they also reported greater physical pain when birthing in the hospital as compared to  at home or in their community. |
| 1371 | Webber and Wilson, 1993. | Childbirth in the north. A qualitative study in the Moose Factory zone. | Qualitative, design using semi-structured interviews and content and thematic analysis with 24 Cree women from the Moose factory Zone. | Ontario, Canada, Indigenous Women. | To document how Cree women perceived evacuation to hospital for birthing. | Traditional research methods including semi-structured interviews. | Women interviewed experienced loneliness, boredom, distressed by separation from family, in some cases depression. | Women showed preference for being accompanied by partner. Many of participants preferred to birth in their local community. |
| 1368 | Watson et al., 2002. | The maternity experiences of indigenous women admitted to an acute care setting. | Descriptive qualitative study of n=12 Indigenous women who birthed at or prior to arrival at Royal Darwin Hospital. | Darwin, Australia, Aboriginal and Torres Strait Islander women. | The aim of this study was to inform patient and professional educational programs to enhance birthing experiences of Indigenous women. | Traditional research methods were employed. Interviews were conducted using informal chat style. Data were analysed using interpretive methods. | Feelings of loneliness and boredom were experienced. Participants reported inadequate interactions with staff, were frightened and described miscommunication, lack of empathy and misunderstanding of cultural and spiritual beliefs. | The women described negative experiences including the need for explanations regarding the birthing experience. |
| 1324 | Varcoe et al., 2013. | Help bring back the celebration of life: a community-based participatory study of rural Aboriginal women's maternity experiences and outcomes. | Participatory action research utilising ethnography. Aboriginal women.  127 parents and family members. | British Colombia, Canada, interviews with over 100 Aboriginal women who had given birth in the last three years as well as fathers. Focus groups were also conducted. | To understand rural Aboriginal women's' experiences of maternity care and factors shaping those experiences. | Critical ethnography withing a participatory action research framework. Traditional analysis methods. | Most participants described distressing experiences during pregnancy and birthing including healthcare professionals who lack understanding of historical and ongoing colonial relations which impact choice and affect birth outcomes. | In addition to geography, prior negative experiences and discrimination are deterrents to accessing prenatal care. |
| 1318 | Vang, et al., 2018. | Interactions Between Indigenous Women Awaiting Childbirth Away from Home and Their Southern, Non-Indigenous Health Care Providers. | Qualitative, participatory research using semi-structured interviews with n=25 Inuit and Indigenous women who were medevacked to northern Quebec for maternity care. | Quebec, Canada, both Inuit and Indigenous women. | To evaluate the impact on Indigenous women medevacked to a large hospital for birth from Northern Quebec.  To explore how  the process of childbirth evacuation is implicated in the  quality of the medical encounter between Indigenous  women and their southern, typically non-Indigenous  health care providers. | Traditional research methods using Grounded Theory and including both semi structured interviews, and open coding using VERBI Software 2017. | Main issues revealed in this study were the social disconnect and social isolation experienced. This study highlighted the need for institutions to instigate cultural sensitivity training that highlights the larger historical, social and political issues experienced by Indigenous women. Findings show that the quality of the patient and provider interaction is contingent on individual health care providers’ ability to connect  with Indigenous patients and overcome cultural and institutional barriers to communication and trust-building. The  findings point to the need for further training of medical professionals in the delivery of culturally safe care and addressing bureaucratic constraints in the health care system to improve patient and provider communication and overall  relationship quality. | Childbirth evacuation is a stressful experience for Indigenous women but separation from families, especially older children was a source of stress. Consideration should be given to use of an escort to accompany woman and acknowledging stress; also, hospital bureaucracy including waiting times, the feeling of being rushed and lack of time to build relationships with staff create stress. Finally, the issue of perceived cultural stereotypes, labelling, communication issues and medical mistrust. |
| 1219 | Simmonds et al., 2012. | The role of support person for Ngaanyatjarra women during pregnancy and birth. | Participatory research with interviews of n=36 Aboriginal women from Western Australia including n=15 older women and n=8 younger women and n=13 young women. | Australia, Aboriginal Ngaanyatjarra women from Western Australia. | To enable the provision of culturally appropriate services to Ngaanyatjarra women. | Participatory research methods using unstructured interviews or bush meetings with older women. Thematic analysis of the findings. | Traditional role of support person for women transferred for hospital birth persist but younger women preferred midwife support. This highlights the changing cultural preferences in central Australia. | Preference for birthing on country model of care to combat feelings of loneliness, disruption to family unit and inability to share traditional knowledge. Presence of a family member during interactions with healthcare professionals may prevent misunderstandings. Birthing women require choice in support person during birth. |
| 1108 | Reibel et al., 2015. | Young Aboriginal women's voices on pregnancy care: factors encouraging antenatal engagement. | Qualitative data collected using yarning with n=28 pregnant women or young mothers and n=36 senior women (total of 64 women) and n=20 service providers (12 Aboriginal and 8 non-Aboriginal). | WesternAustralia, a mix of cultures including Aboriginal and non-Aboriginal participants. | To understand young Aboriginal women's views on pregnancy care. | Data was collected using a bi-cultural approach and semi-structured yarning, and data interpretation by firsthand cultural knowledge and current evidence. Employment of Aboriginal cultural consultant as researcher. Constant comparative analysis. | Importance of family support and reports of loneliness and isolation when relocated to tertiary setting. Importance of role of female family members in directing engagement in antenatal care. | There is a strong cultural connection for the whole family when baby is due. Relocation requirements can result in resistance to attend antenatal care and disengagement from pregnancy care. |
| 1046 | Parker et al., 2014. | 'Choice, culture and confidence': key findings from the 2012 having a baby in Queensland Aboriginal and Torres Strait Islander survey. | Quantitative and qualitative data were obtained from both online and hard copy survey of n=187 Aboriginal and Torrens Strait Islander women who had birthed in Queensland in 2011 and 2012. | Queensland, Australia, Aboriginal and Torrens Strait Islander women. | To describe the maternity care experiences of Aboriginal and Torres Strait Islander women birthing in hospitals in Queensland. | Traditional research methods including survey and data analysis using SPSS (version21) and thematic analysis of qualitative data. Use of Aboriginal and Torres Strait Islander peer interviewers. | Participants valued continuity of care, but cited incidences of being ignored, talked down to, having limited choice in care or choose gender of care provider. | Strong preference to deliver baby in own community. Inability to carry out any cultural practices during pregnancy and birth and strong need for cultural competence training amongst healthcare providers. Strong need to value family support and improves women's experiences of care. |
| 1040 | Pandey et al., 2023. | Indigenous birth support worker (IBSW) program evaluation: a qualitative analysis of program workers and clients' perspectives. | Qualitative, focus groups with Indigenous Birth Support workers (n=4) and interviews with n=10 clients, written feedback from n=3 clients. | Canada, Indigenous birth support workers and Indigenous clients of the program. | The clients of the service greatly appreciated and respected the IBWS cultural support and the compassionate non - judgemental and culturally safe care. | Traditional research methods including interviews, focus groups and thematic analysis. | Improved client centred, trauma informed care, improved pain management and enhanced relationships with community Elders. IBSW were able to identify patients, physical, emotional cultural and spiritual needs. | Healthcare insensitivity towards Indigenous clients persists. There is a need for greater collaboration with healthcare practitioners and enhanced evidence-based practice. Lack of family support whilst hospitalise remains an issue. Strong need for IBSW integration into healthcare system including prenatally and need for culturally safe training for all providers. |
| 1033 | Paberzyte, 2019. | Continuity and change in Wemindji Cree childbirth experiences and practices: Past and present. | Qualitative, ethnographic study employing interviews with 36 Indigenous mothers, elders and 16 medical staff. | Quebec, Canada, Wemindji, Cree women. | To explore birth experiences of Wemindji, Cree women. | Traditional research methods of anthropology and ethnography with no discussion of analysis methods. | The Cree accepted the evacuation for birth policy as a strategy of superficial compliance. | Cree women showed a strong preference for birthing in the community. The lack of family and community were issues in hospital births. |
| 937 | Montgomery-Andersen et al., 2010. | 'There was no other way things could have been.' Kalaallit Nunaat women's experiences of referral and transfer during pregnancy. | Narrative methodology including interviews with n=9 Kalaallit Nunaat women. | Kalaallit Nunaat | To evaluate birthing experiences of Kalaallit Nunaat women giving birth in an urban referral centre. | Traditional research methods using narrative methodology and interviews. Analysis methods are not discussed. | Women valued the importance of cultural safety, family supports. | Cultural safety issues existed along with feelings of isolation from community and family and lack of purpose. |
| 887 | McCalman et al., 2024. | "Safe, connected, supported in a complex system." Exploring the views of women who had a Indigenous baby at one of three maternity services offering culturally tailored continuity of midwife care in Victoria, Australia. A qualitative analysis of free-text survey responses. | Descriptive exploratory method using survey and interviews employing a Critical Race Theory Lens.  N=343 and n=213 follow up questionnaire. Participants, identified with 60 First Nations communities from across the Australian continent. | Australia, Aboriginal women birthing in urban hospitals. | To explore the experiences of women birthing a Indigenous baby in three major hospitals in Melbourne which employ Midwifery continuity of care. | Use of traditional methods underpinned by a Critical Race Theory Lens. | Women valued the continuity of care model, felt emotionally supported and that the care they received was personalised to their needs, but other issues persist. | Women experienced cultural safety issues, fragmented care and some staff who did not listen and were unsupportive. |
| 740 | Kornelson et al., 2011. | Alienation and Resilience: The Dynamics of Birth Outside Their Community for Rural Indigenous Women. | Quantitative written survey data of n=35 Aboriginal women who gave birth away from their communities. Interviews with n=3 women who gave birth away from their community. | Canada, Aboriginal women. | Women who gave birth away from their rural communities expressed feelings of | Traditional research methods including survey, interview and thematic analysis. | Women reported feeling powerless in the urban birthing environment. | Women reported a preference for delivering in community due to travel issues, feelings of isolation, missing family, and estrangement from cultural norms. |
| 656 | Josif et al., 2013. | 'No more strangers': Investigating the experiences of women, midwives and others during the establishment of a new model of maternity care for remote dwelling aboriginal women in northern Australia. | Mixed methods design within a Participatory Action Research, framework, n=6 Midwifery group practise midwives, n=2 Aboriginal health workers, n=34 other staff, 12 Aboriginal women who used the service and n=1 senior Aboriginal woman. | Northern Territory, Australia, Aboriginal women and staff working in the Northern Territory. | Some women perceived being relocated to central service as being dragged away from their families. | Traditional research methods using semi structured interviews, field notes and thematic analysis. | Lack of continuity of care was identified as a major factor. | Positive impact of the Midwifery Group practice midwives on the culture and responsiveness of the wider maternity service because of their knowledge of women's backgrounds provided insight into remote dwelling women's circumstances. |
| 592 | Ireland et al., 2011. | Niyith Nniyith Watmam (the quiet story): Exploring the experiences of Aboriginal women who give birth in their remote community. | An ethnographic study of birth histories of Aboriginal women and family members in the NT who refused transfer to urban hospitals prior to birth.  N=7 Aboriginal women and n=5 family members. | Australia, Northern Territory and Aboriginal women and family members. | To collect birth stories, beliefs and practices of Aboriginal women in the community, many who had experienced transfers to urban hospitals for previous births. | Traditional research methods including ethnography and manual thematic analysis. However, ˜message sticks” were sent to potential participants via word of mouth about the research to women in the community. | Recommendations include a re-establishment of community birthing in the Saint Gerard region to avoid. Whilst this is not an evaluation of a hospital birthing service some of the findings may be relevant to this review. | The women interviewed saw the current model of maternity care that involves transfer for a planned hospital birth as associated with infringement of Women’s Business Laws. According to Aboriginal Law, all matters relating to reproduction are ˜Women’s Business. Although the boundaries of Women’s Business are changing, the narratives described experiences in Darwin that highlighted the dislocation of women from their culturally appointed carers, and the lasting emotional impacts of shame and technological violation during childbirth. The presence of male health care providers continues to be a significant concern for Aboriginal women. |
| 350 | Dietsch et al., 2010. | Australian Aboriginal kinship: a means to enhance maternal well-being. | Qualitative design utilising interviews with three Aboriginal women and one partner as well as field notes. Thematic analysis. | Wagga Wagga, NSW, Australia. Aboriginal mothers. | Aboriginal women are impacted by rural closures of maternity services in NSW but the effects of this are mitigated by support from kin. | Traditional research methods including In- depth interviews, and analysis of field notes whilst utilising the experience of an Aboriginal midwife. Thematic analysis employed. | Aboriginal women experience feelings of loneliness, fear, isolation and alienation when forced to relocate to large city hospitals to deliver. They felt disrespected, oppressed culturally unsafe and experienced racism. | Aboriginal women’s ties to country (birthing on country) and kinship networks should be valued and models of midwifery care developed to ensure healthy pregnant women have a choice as to whether they remain on country or transfer, from their country and kin, to birth. |
| 290 | Churchill et al., 2020. | Conceptualising cultural safety at an Indigenous-focused midwifery practice in Toronto, Canada: qualitative interviews with Indigenous and non-Indigenous clients. | Qualitative interviews with former clients of seventh generation midwives Toronto (SGMT), n=20 including n=9 indigenous and n=11 non-indigenous participants. | Toronto, Canada, both Indigenous and non-indigenous participants. | To explore how clients conceptualise and experience cultural safety. | Traditional methods used including purposive sampling, interviews and thematic analysis. Co-led by Indigenous midwives and analysis conducted using a methodological approach to centre Indigenous perspectives true to the lived experiences of Indigenous and Non-Indigenous participants. | Cultural safety was conceptualised as having personalised continuous relationships with midwives. Having a space that made participants feel "at home". | Indigenous and non-indigenous participants conceptualised and experienced cultural safety in different ways. |
| 222 | Campbell et al., 2004. | Maternity care with the women's business service at the Mildura Aboriginal Health Service. | Qualitative, face to face interviews with n=25 Aboriginal and non-Aboriginal women. Programme evaluation. | Mildura, Australia, Aboriginal women. | Women using the Women’s Business Service were significantly more positive about many aspects of their care than women attending other rural public maternity services. The study lends support to the view that Aboriginal community- controlled health services are well placed to provide appropriate and accessible care to Indigenous women. | Traditional methods used for data collection -face to face interviews and quantitative analysis and comparison of findings to an earlier study. | Women participating in the Mildura WBS evaluation were significantly more positive about many aspects of their care in pregnancy and postnatally than women attending other rural public maternity services. They were less happy with intrapartum and early postnatal care in hospital. | Aboriginal community-controlled health services are well placed to provide appropriate and accessible care to  Indigenous women during pregnancy and the postnatal period. |
| 193 | Brown et al., 2019. | Health care experiences and birth outcomes: Results of an Aboriginal birth cohort. | Population based study of women giving birth in South Australia, n=344, face to face interviews or structured questionnaire with Aboriginal researcher. 83% elected to complete the questionnaire. | South Australia, Aboriginal women giving birth to an Aboriginal baby between June 2011 - July 2013. | Discrimination was more likely to be reported by women experiences three or more stressful events or social health issues. | Consultation with Aboriginal community and use of Aboriginal researcher but traditional research methods used. | 51% of women reported receiving discrimination or unfair treatment from hospitals or health services during pregnancy or shortly after. | Aboriginal women most at risk of poor infant health outcomes were the least likely to perceive that they received care well matched to their needs. |
| 187 | Brown et al., 2016. | Aboriginal and Torres Strait Islander women's experiences accessing standard hospital care for birth in South Australia - A phenomenological study. | Qualitative, Interpretive Heideggerian/phenomenological design. Semi-structured interviews with 14 Aboriginal and Torrens Strait Islander women. Thematic analysis conducted on data. | South Australia, Aboriginal and Torres Strait Islander mothers who chose to give birth in a large tertiary maternity teaching hospital in South Australia. | Six main themes including: knowing what is best and wanting was best for my baby; communicating my way; how they made me feel; all of my physical needs were met; we have resilience and strength despite our hardships; and recognising my culture. | Phenomenology was used as a data collection method as it reflects the Aboriginal storytelling and Yarning culture which is a culturally safe method of sharing information. | Positive outcomes came when staff practised open inclusive communication with the women. Feelings of being judged by midwives negatively impacted their experience. Many experienced isolation and hardship in the need to relocate to the city. All participants stressed the benefits of a culturally safe approach. Participants disliked being judged due to culture, excluded from decision-making related to their care. They felt isolated and alone. | The critical finding of this study is that culturally safe care, emphasizing strong communication and active decision-making involvement, is essential to improve childbirth experiences for Aboriginal and Torres Strait Islander women, who often face judgment and cultural misunderstandings. |
| 18 | Adcock et al., 2021. | He Tamariki Kokoti Tau: Families of Indigenous Infants Talk about Their Experiences of Preterm Birth and Neonatal Intensive Care. | Cross-sectional interpretative phenomenological analysis of interviews with 19 whanau (family collectives) participating in a Kaupapa Māori (by, with, for Māori) qualitative longitudinal study of preterm birth identified themes from their experiences and the meanings they attributed to them.  19 mothers. | Wellington, Aotearoa (New Zealand). Māori (Indigenous peoples of Aotearoa). | Examined the experiences of Māori whanau to give voice to their experiences, as they journeyed along preterm care pathways, from birth for one year.   To support service transformation to ensure that whanau are supported as they face the joys and challenges of parenting their precious newborn gift. | Kaupapa Māori (by, with, for Māori).  Data were analysed using interpretative phenomenological analysis (IPA), which allows for the bottom-up emergence of themes related to participants experiences and the meanings they attribute to them. | ˜An Emotional Roller Coaster”. Disruption. Self-Doubt and Guilt. Fear for and of Fragile Infants.  ˜It Does Get Quite Lonely Sometimes”. Isolation. Inhospitable Spaces. Lack of Autonomy. The Importance of Familiarity When â€˜Family Is the Best Support Network Most of Us Have. Importance of Whanau and Peer Support. Culturally Safe Care. | Intergenerational relationships must be included in care. Whanau are experts in their own worlds and of their infants, and ought to be listened to and supported to find the intimacy with their infants that gives them strength. For whanau to feel at home, for the chaos to quieten, they should be wrapped up in love, by wider whanau, peers, and health practitioner champions. These factors contribute to whanau well-being and should be prioritised to inform  Māori-centred models of neonatal care.  The critical finding of this study is that intergenerational relationships, whanau expertise, and the provision of love and support from whanau, peers, and health practitioners are essential for enhancing whanau well-being and should be central to Māori-centred models of neonatal care. |
| Google scholar | Brown & Fiske 2001. | Indigenous Women’s encounters with mainstream health care services. | Qualitative design employing Critical feminist ethnography incorporating in-depth interviews with 10 women including use of field notes. | Northwestern Canada, Indigenous women. | Sought to gain an understanding of Indigenous Women’s encounters with mainstream health services. | Data were analysed using interpretive thematic analysis. | The women reported negative stereotypes about Indigenous Women. Some reported affirming encounters with respect shown for cultural identity. | Need for cultural safety training to ensure Western nurses challenge cultural assumptions about Indigenous Women. |
